# Incidental Finding of Thickened Endometrium in Postmenopausal Women: A Survey of Endometrial Cancer

**DOI:** 10.7759/cureus.38538

**Published:** 2023-05-04

**Authors:** Michela Quaranta, Katherine Maillou, Natasha D'Souza, Pubudu Pathiraja

**Affiliations:** 1 Gynecological Oncology, Addenbrooke's Hospital, Cambridge University Hospitals NHS Foundation Trust, Cambridge, GBR; 2 Obstetrics and Gynecology, Addenbrooke's Hospital, Cambridge University Hospitals NHS Foundation Trust, Cambridge, GBR

**Keywords:** postmenopausal bleeding, investigation increased endometrial thickness, endometrial cancer, atypical hyperplasia of endometrium, incidental finding increased endometrial thickness

## Abstract

Objectives

The primary objective was to determine the prevalence of endometrial cancer in asymptomatic and symptomatic postmenopausal women referred to the hysteroscopy service for incidental finding of thickened endometrium. The secondary objectives were to identify, for the asymptomatic cohort, an acceptable threshold of endometrial thickness (ET) which should trigger endometrial sampling and its related sensitivity and specificity.

Methods

This was a retrospective cohort study of 136 asymptomatic and 602 symptomatic postmenopausal women with an ET of >4 mm referred to the endometrial cancer diagnostic service in a gynecology oncology center over a period of one year. Clincal and demographic data were analyzed. Histopathological diagnosis was completed and receiver operating characteristic (ROC) curves for acceptable ET cutoff in asymptomatic women were evaluated.

Results

The prevalence of malignancy and atypical hyperplasia in asymptomatic women was 3.7% and 4.4%, respectively. Within the asymptomatic subgroup with ET <11 mm, the yield rate for atypical hyperplasia (AH)+cancer was 2.2%. An ET <10 mm gave a similar yield; however, specificity decreased. ET (t-test p-value=0.037) correlates with endometrial pathology. Receiver operating characteristic (ROC) curves identified a cutoff of 11 mm as an acceptable threshold for triggering further investigations.

Conclusion

Based on our findings, 11 mm may represent an acceptable threshold for further investigation in asymptomatic postmenopausal women. We strongly advocate qualitative assessment of the endometrium and evaluation of individual risk factors in women with ET between 4 mm and 11 mm. This study will contribute to the existing body of evidence for the management of asymptomatic postmenopausal women with incidental increased ET. Further studies are required.

## Introduction

Endometrial cancer (EC) is the most common gynecological malignancy in the developed world. Rates have increased over the past 50 years, with some studies estimating this as high as 56% [1]. Obesity and longer life expectancy are cited as the main contributing factors [2]. Uterine cancer is responsible for 5% of all new cancer diagnoses in females and represents the fourth most common cancer in females in the United Kingdom with 9700 new cases annually [3]. The majority of women diagnosed with endometrial cancer experience bleeding [4-6]. It is unknown how many asymptomatic women are diagnosed with EC; however, these may represent 5-20% [5]. In the United Kingdom, women who experience postmenopausal bleeding have a clear pathway of a two-week wait (2WW) referral with pelvic ultrasound [7]. The British Gynecological Cancer Society uterine cancer guidelines (BGCS) 2021 suggest a cutoff of 4 mm [8], based on a large meta-analysis by Smith-Bindman et al. in 2004, for further assessment with hysteroscopy and biopsy [5]. It is widely agreed in literature that an endometrial thickness of <4 mm carries a low risk of endometrial cancer [2,5,8].

The wide availability of imaging has led to inadvertent screening with an increase in the number of asymptomatic women referred. A recent review estimates prevalences of endometrial carcinoma and atypical endometrial hyperplasia in this population are 0.62% and 0.59%, respectively [7]. Thus, others have suggested that applying evidence and criteria from postmenopausal bleeding data to an asymptomatic population is not justifiable in view of the low overall disease prevalence and poor performance of transvaginal ultrasound scan (TVS) in detecting serious endometrial disease at all cutoffs [9]. International societies recommend a cutoff rate of 11 mm as acceptable for further diagnosis [10,11]. These recommendations are based mainly on the data of Hefler et al. and Smith-Bindman et al. which demonstrate a risk of endometrial cancer in asymptomatic women with an endometrial thickness ≥11 mm at 5.9% [4,5]. However, clinical guidelines in the United Kingdom do not address the issue and there are currently no guidelines in place. This leads to variation in practice and potential of over-treatment with an impact on both the women and on the service provider [12]. This has become particularly relevant with the introduction of the faster diagnostic strategy (NHS Long Term Plan 2019) and the impact of coronavirus disease 2019 (COVID-19) [13].

Our study aimed to evaluate and compare the prevalence of atypical hyperplasia (AH) and endometrial cancer in asymptomatic vs symptomatic women. Furthermore, we aimed to determine an optimal cutoff for further diagnosis and to analyze the impact of these interventions in terms of failure/complication rates. To our knowledge, this is the first study of its kind in our region.

## Materials and methods

Study design

This was an observational single-center retrospective study conducted in a tertiary referral cancer center in the East of England (Addenbrooke’s Hospital, Cambridge, UK) from March 1, 2019, to March 1, 2020. 

Data collection

Eligible consecutive women referred to our Gynecology 2WW pathway with postmenopausal bleeding or an incidental finding of increased endometrial thickness over the study period were included. Women with an endometrial thickness (ET) ≥4 mm, regardless of symptoms, were offered diagnostic hysteroscopy and endometrial sampling as office-based blind endometrial sampling is not routinely offered in our unit. In the event of refusal, follow-up imaging was performed, and if ET was stable women were discharged. The endometrial thickness was measured by TVS as the thickest part in the sagittal plane of the uterus and recorded as a single layer. The presence of fluid or irregular lesions and vascularity were noted according to the International Endometrial Tumor Analysis (IETA) group. Exclusion criteria were premenopausal status, a diagnosis of Lynch, and use of hormonal replacement treatment (HRT) or tamoxifen as these may impact the appearance of endometrium on ultrasound.

A total of 1010 patients were referred to the service during the study period. Of the 738 eligible women, 136 (18.4%) were asymptomatic. Epidemiological and clinicopathological data were collected retrospectively from existing electronic patient records and included the following: age, menopausal status, parity, body mass index (BMI), presence/absence of symptoms, ultrasound findings (endometrial thickness and qualitative description), hysteroscopic impression, and histology. All samples were reviewed by center pathologists and recorded as atrophic endometrium, benign polyp, atypical hyperplasia (AH), or endometrial cancer. All cases of AH or cancer were reviewed and discussed at the cancer multidisciplinary meeting. Although not the primary remit of the study, data regarding outpatient "failure" rates and operative complications were also recorded. The incidental findings cohort was further assessed to determine an ideal ET threshold for further diagnosis. Patients were subsequently stratified and analyzed according to this threshold. All patients in the asymptomatic group were followed up for a period of approximately 24 months. The National Health Service (NHS) Caldicott principles were strictly adhered to in all data collection and handling [14]. The study was approved as a service evaluation by the Trust’s clinical governance committee.

Statistical analysis

Statistical analyses were performed with SPSS version 20 (Chicago, IL: SPSS Inc.). Continuous variables were compared using Student’s t-test. Potential confounders were identified using the non-parametric χ^2^ or Fisher’s exact test. Multiple parameter analyses were performed using binary logistic analysis calculating odds ratio (OR) at 95% confidence intervals (95% CI). Receiver operating characteristic (ROC) curve analysis was used to determine the cutoff value of ET for AH+cancer by area under the curve (AUC).

## Results

**Figure 1:**
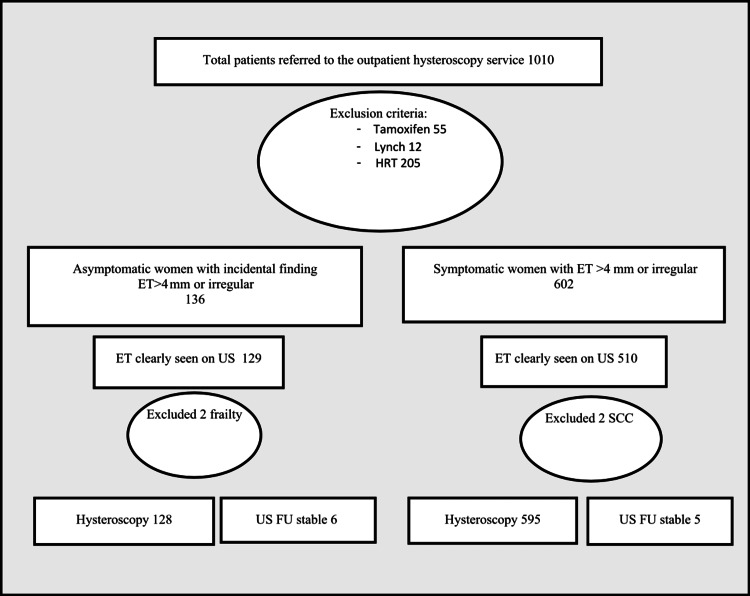
Flow diagram of cohort selection and application of exclusion criteria. Exclusion criteria include the use of hormone replacement treatment (HRT) tamoxifen and women diagnosed with Lynch syndrome. Hysteroscopy was offered to all women; if declined then ultrasound follow-up (US FU) was performed, and if endometrial thickness (ET) was stable women were discharged. Two women in the asymptomatic group did not undergo any investigation due to poor performance status and two women in the symptomatic group were excluded due to diagnosis of squamous cell carcinoma (SCC) of the cervix.

A total of 1010 records were reviewed. After exclusion criteria, 738 women were included of which 136 for incidental findings on imaging which formed the sole indication for referral (Figure 1).

All women included were postmenopausal. The age varied from 47 to 99 years. The mean age in the asymptomatic group was significantly higher than in the symptomatic group (70.3 vs 61.5 years) and body mass index was not dissimilar in the two groups. The characteristics of the study population are described in Table 1 with further specific risk factors being assessed only in the asymptomatic cohort to assist with decision-making.

**Table 1:** Demographic characteristics of the entire population - risk factors for EC were collected only for the asymptomatic group. HTN: hypertension; EC: endometrial cancer

Variables	Symptomatic (602 women)	Asymptomatic (134)
Age	61.54 (54-89)	70.3 (47-99)
BMI	29.5 (16.1-61.5)	28.6 (14.7-49)
Nulliparous	-	12 (8.9%)
Diabetes	-	13 (9.7%)
HTN	-	62 (46%)

All women were offered hysteroscopy with 595 women in group A and 128 women in group B accepting this procedure. The women who declined underwent interval ultrasound follow-up at 12 weeks and if the ET remained stable, they were discharged. The majority of procedures were performed in an outpatient setting; however, the requirement for general anesthetic was higher in the asymptomatic group (25% {32 women} vs 15.6% {93 women}). Complications occurred only in the symptomatic group (three false passages and one uterine perforation); however, the asymptomatic women had a higher failure rate due to cervical stenosis.

Pathological findings are listed in Table 2. Six (4.4%) women in the asymptomatic group were diagnosed with AH vs 18 (2.9%) women in the symptomatic group. A total of five cases of cancer were identified in the asymptomatic group and 47 in the control group (Table 2). We also noted a higher prevalence of polyps in the asymptomatic cohort.

**Table 2:** Details of pathology in the study groups. Other histology includes two cases of metastatic cervical cancer in the symptomatic group and one case of metastatic breast cancer in the asymptomatic group.

Histology	Symptom	%	95% CI	No symptom	%	95% CI
Benign	532	88.4%	-	117	86%	-
1. Atrophic endometrium	388	64.4%	0.605-0.683	61	44.8%	0.363-0.536
2. Polyps	144	23.9%	0.206-0.275	56	41%	0.328-0.499
Atypical hyperplasia	18	3%	0.018-0.047	6	4.4%	0.106-0.094
Cancer	47	7.8%	0.0-0.102	5	3.7%	0.012-0.084
1. Low grade	26	4.2%	-	1	0.7%	-
2. High grade	19	3.3%	-	3	2.2%	-
3. Other	2	0.3%	-	1	0.7%	-
Failed	5	0.8%	-	6	5.9%	-
Total	602	100%	-	136	100%	-

All patients had an ET of >4 mm or irregularities as defined by IETA as irregular outline, intracavitary lesion, or abnormal vascularity. Average ET is reported in Table 3. An increased average ET was observed in the group of AH+cancer compared to benign histology in both the symptomatic and asymptomatic groups. This difference was statistically significant in the asymptomatic group (Student's t-test, p=0.03). A positive non-significant association between bleeding and endometrial pathology (OR: 1.34; 95% CI: 0.693, 2.640) was observed. No statistical difference in endometrial thickness when endometrial malignant pathology (AH and cancer) was diagnosed (15.48 mm vs 14.1 mm) in the symptomatic vs asymptomatic group (p=0.637). On the contrary, a statistically significant difference in endometrial thickness was observed between the benign and AH+cancer subgroups within the asymptomatic population (Student's t-test, p-value=0.03). Five women in the asymptomatic group and six in the symptomatic group had ultrasound follow-ups. Average ET was 5.1 mm (range: 4.2-7.4 mm) in the asymptomatic group and 8.8 mm (range: 5-14 mm) in the symptomatic group.

**Table 3:** ET (in mm) measured in benign and AH and cancer diagnosis in both groups of women where endometrium clearly visible. ET: endometrial thickness Four women from the asymptomatic group and 12 from the symptomatic group were excluded as endometrium was not clearly identified.

Variables	Asymptomatic women mean ET±SD (mm)	Symptomatic women mean ET±SD (mm)
Benign endometrium	9.6±6.3	7.7±4.8
Atypical hyperplasia	14.2±5.5	14.3±5.4
Cancer	13.6±4.7	15.5±9.4

Cutoff values of endometrial thickness as a predictor of pathology

**Figure 2:**
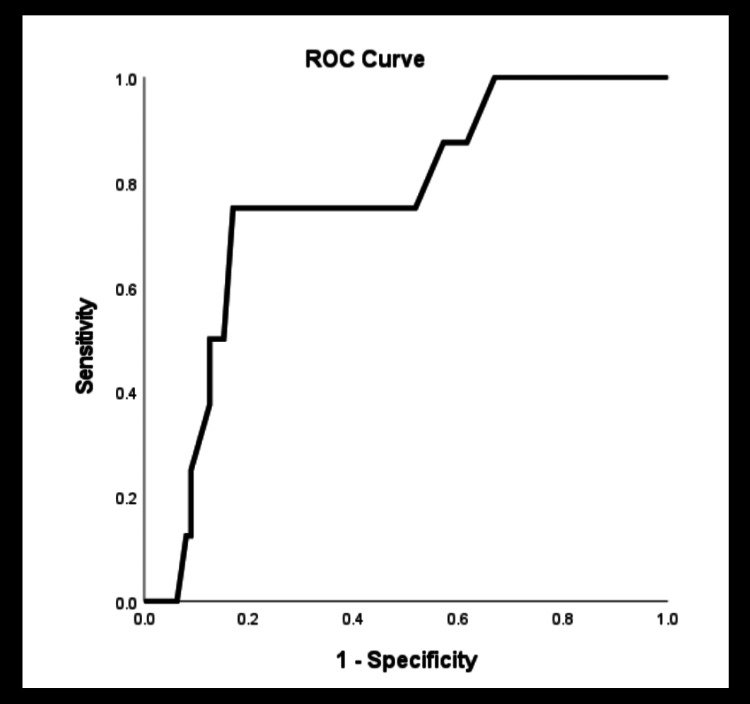
Endometrial thickness receiver operator curve characteristic (ROC) for atypical hyperplasia and cancer. At an ET of 11 mm, AUC is 0.762. ET: endometrial thickness; AUC: area under the curve

Figure 2 shows the ROC curve for the estimated diagnostic performance of TV ultrasound measurements in detecting AH/endometrial cancer. In ROC analysis, endometrial thicknesses of 10, 11, and 12 mm all had sensitivities of 75% and specificities of 67%, 76%, and 75%, respectively. The relatively poor sensitivity noted demonstrates the importance of taking into consideration qualitative findings of the ultrasound and not relying solely on ET. The single case of cancer with ET <11 mm had ultrasound features suggestive of malignancy (vascularity, irregularity, hyperechoic mass) and likely would not have been missed.

The yield rate of cancer and atypical hyperplasia was 3.7% and 4.4% in the entire asymptomatic population (n=134). When considering an ET<11 mm, the yield rate of atypical hyperplasia (AH) and cancer was 1.1% and 1.1%, respectively (total: 2.2%). This remained similar when considering a cutoff of 10 mm; however, specificity decreased. Table 4 demonstrates the outcome of investigations using a threshold of 11 mm combined with features of suspicion on ultrasound. This achieved a diagnostic sensitivity of 90.9% (95% CI: 59-100) and specificity of 64.8% (95% CI: 56-73) for cancer/atypia.

**Table 4:** The outcome of investigations when using an ET >11 mm and ultrasound features of suspicion. ET: endometrial thickness; AH: atypical hyperplasia

Variables	ET <11 mm and no suspicious features	ET >11 mm or suspicious features	ET not clearly visible or irregular/suspicious features
Benign endometrium	57 (71.4%)	12 (26.6%)	1 (25%)
Endometrial polyp	23 (26.4%)	31 (68.8%)	0
AH+cancer	2 (2.2%)	7 (15.5%)	2 (75%)
Total	87	45	3

Risk factors for AH and/or endometrial cancer

We further analyzed risk factors for EC to identify the impact of risk factors on our population. Comparisons between asymptomatic patients with benign results and AH/endometrial cancer are listed in Table 5. In univariate analysis, age was the only factor associated with endometrial pathology (cancer/AH mean age 74 years SD 5.2 vs benign mean age 70.8 years SD 10.5, p=0.045). This is unexpected considering that 27% of all uterine cancers are diagnosed in women aged 75 years or over with the highest incidence reported in the 75-79 years age group in the United Kingdom [3]. The sample size is small however and must be interpreted with caution.

**Table 5:** Baseline characteristics within the asymptomatic cohort. AH: atypical hyperplasia

Characteristics	Benign	AH+cancer	p-Value
Age	70.8±10.5	74±5.2	0.045
Nulliparous	10% (11)	11% (1)	0.275
BMI	29.4±7.8	29.74±7.4	0.601
Diabetes	9% (10)	22% (2)	0.736
Hypertension	47% (53)	40% (5)	0.736

The asymptomatic patients were followed up till May 2022 (approximately 24 months). Seven women were returned to the clinic for the following reasons: two for bleeding, two for abnormal discharge, and three women for suspicious endometrium on imaging. All women underwent hysteroscopy and biopsy. Histology was benign in all cases.

## Discussion

This study of postmenopausal women with thickened endometrium has shown a lower prevalence of pathology in asymptomatic compared to symptomatic women (8.1% vs 10.8%). This was notwithstanding the average older age group and consequent higher baseline risk in the asymptomatic population (70.3 years vs 61.5 years in symptomatic cohort). This was also expressed by Felix et al. in the PLCO trial and the majority of cancers are diagnosed in symptomatic women as widely reported in the literature [15,16].

The rates of endometrial cancer observed are consistent with those reported by the large-scale FAME-Endo trial [4] and by studies of similar size by Aggarwal et al. (3.6%) and Ghoubara et al. (3.7%) [17,18]. Of note our study differs in strict exclusion criteria with HRT and tamoxifen users excluded and this may account for slight variations. Interestingly, we observed higher rates of atypical hyperplasia within the asymptomatic group (4.4%). This is not reflected in literature although most studies did not directly compare data and may be explained by patient selection bias at source. However, further large-scale investigation is required. It is important to stress that there is no reported survival advantage in diagnosing AH or cancer before symptoms occur and it may well be that these women would have gone on to develop symptoms [15,18,19].

The high rates of polyps seen in our population vary with endometrial thickness and are not dissimilar to those reported by Lee et al. (23.1%) and Schmidt et al. (73.3%) when ET cutoff is applied [20,21]. Endometrial thickness in asymptomatic women was shown to correlate with pathology (p=0.03) as is the case in symptomatic women. The British Gynecological Cancer Society (BGCS) 2021 [8] and the British Medical Ultrasound Society (BMUS) [21] however caution against applying the same endometrial thickness cutoff to asymptomatic patients. As prevalence in this group is low, the index of suspicion should be high to warrant further invasive testing [22]. Most existing recommendations quote a 10 mm or 11 mm cutoff as a trigger for endometrial sampling. This is based on the work by Hefler et al. in 2018 and Smith-Bindman et al. in 2004 and which report a risk of cancer in asymptomatic women with an ET >11 mm and ET >10 mm to be approximately 6.7% and 5.8%, respectively, (similar to the risk reported for symptomatic women with an ET >5 mm and an ET >4 mm) [4,5].

In our study group, only one patient was diagnosed with endometrial cancer and one patient with AH had an ET of less than 11 mm. This places the risk of endometrial pathology in patients with an ET <11 mm at 2.4%. These findings are similar to those of Aggarwal et al. and Famuyide et al. who examined similar-sized cohorts of asymptomatic women, although unlike ours, women on HRT and tamoxifen were not excluded from their study [17,22]. Calculated ROC curves show minimal difference in considering cutoff values of 10, 11, or 12 mm with a slightly lower specificity seen at 10 mm. Although not a focus of the study, the qualitative description of the endometrium merits comments as irregularity and vascularity are more likely to be associated with pathology. The single case of cancer with ET <11 mm was described as irregular and suspicious and all cases of AH were identified within polyps.

The safety of our findings is reinforced by the fact that none of the patients in the study cohort developed either preinvasive or invasive disease in the following 24-month period. The strengths of this study include recruitment of consecutive patients over a 12-month period, exclusion of HRT and tamoxifen users, and a 24-month follow-up period. Limitations include single-center site, small sample size, and low prevalence of disease. Furthermore, confounding factors were only evaluated for the study group. The impact of these however is well documented in literature and is unlikely to have changed outcomes. The small numbers of pathology detected may explain certain unexpected outcomes and larger studies are warranted.

## Conclusions

The same radiological finding carries different significance in different populations. We cannot ignore underlying patient risk factors and prevalence of disease in the context of a screening test. Based on our findings and in comparison with the widely accepted threshold for symptomatic women, in agreement with both the SCOR and BMUS, we feel that 11 mm represents an acceptable threshold for further investigation in asymptomatic postmenopausal women. However, prospective multicentric studies are required to assess the safety of the threshold. We would strongly advocate qualitative assessment of the endometrium and evaluation of individual risk factors in women between 4 mm and 11 mm.

## References

[1] Yi M, Li T, Niu M, Luo S, Chu Q, Wu K (2021). Epidemiological trends of women's cancers from 1990 to 2019 at the global, regional, and national levels: a population-based study. Biomark Res.

[2] Gentry-Maharaj A, Karpinskyj C (2020). Current and future approaches to screening for endometrial cancer. Best Pract Res Clin Obstet Gynaecol.

[3] (2021). National Cancer Registration and Analysis Services. https://www.ons.gov.uk/peoplepopulationandcommunity/healthandsocialcare/conditionsanddiseases/bulletins/cancerregistrationstatisticseng.

[4] Hefler L, Lafleur J, Kickmaier S (2018). Risk of endometrial cancer in asymptomatic postmenopausal patients with thickened endometrium: data from the FAME-Endo study: an observational register study. Arch Gynecol Obstet.

[5] Smith-Bindman R, Weiss E, Feldstein V (2004). How thick is too thick? When endometrial thickness should prompt biopsy in postmenopausal women without vaginal bleeding. Ultrasound Obstet Gynecol.

[6] Karlsson B GS, Wikland M, Ylöstalo P, Torvid K, Marsal K, Valentin L (1995). Transvaginal ultrasonography of the endometrium in women with postmenopausal bleeding--a Nordic multicenter study. Am J Obstet Gynecol.

[7] (2015). Suspected Cancer: Recognition and Referral. https://www.nice.org.uk/guidance/ng12.

[8] Morrison J, Balega J, Buckley L (2022). British Gynaecological Cancer Society (BGCS) uterine cancer guidelines: recommendations for practice. Eur J Obstet Gynecol Reprod Biol.

[9] Breijer MC, Peeters JA, Opmeer BC, Clark TJ, Verheijen RH, Mol BW, Timmermans A (2012). Capacity of endometrial thickness measurement to diagnose endometrial carcinoma in asymptomatic postmenopausal women: a systematic review and meta-analysis. Ultrasound Obstet Gynecol.

[10] Wolfman W, Leyland N, Heywood M (2010). Asymptomatic endometrial thickening. J Obstet Gynaecol Can.

[11] Aad G, Abajyan T, Abbott B (2013). Search for dark matter candidates and large extra dimensions in events with a photon and missing transverse momentum in pp collision data at sqrt[s]=7 TeV with the ATLAS detector. Phys Rev Lett.

[12] De Silva PM, Carnegy A, Smith PP, Clark TJ (2020). Local anaesthesia for office hysteroscopy: a systematic review &amp; meta-analysis. Eur J Obstet Gynecol Reprod Biol.

[13] England N NHS long term plan. https://www.england.nhs.uk/long-term-plan/#:~:text=As%20medicine%20advances%2C%20health%20needs,Plan%20will%20do%20just%20that..

[14] Crook MA (2003). The Caldicott report and patient confidentiality. J Clin Pathol.

[15] Alcázar JL, Bonilla L, Marucco J, Padilla AI, Chacón E, Manzour N, Salas A (2018). Risk of endometrial cancer and endometrial hyperplasia with atypia in asymptomatic postmenopausal women with endometrial thickness ≥11 mm: a systematic review and meta-analysis. J Clin Ultrasound.

[16] Felix AS, Weissfeld JL, Pfeiffer RM (2014). Endometrial thickness and risk of breast and endometrial carcinomas in the prostate, lung, colorectal and ovarian cancer screening trial. Int J Cancer.

[17] Aggarwal A, Hatti A, Tirumuru SS, Nair SS (2021). Management of asymptomatic postmenopausal women referred to outpatient hysteroscopy service with incidental finding of thickened endometrium - a UK District General Hospital experience. J Minim Invasive Gynecol.

[18] Ghoubara A, Emovon E, Sundar S, Ewies A (2018). Thickened endometrium in asymptomatic postmenopausal women - determining an optimum threshold for prediction of atypical hyperplasia and cancer. J Obstet Gynaecol.

[19] Segev Y, Dain-Sagi L, Lavie O, Sagi S, Gemer O (2020). Is there a survival advantage in diagnosing endometrial cancer in asymptomatic patients? A systemic review and meta-analysis. J Obstet Gynaecol Can.

[20] Lee N, Lee KB, Kim K (2020). Risk of occult atypical hyperplasia or cancer in women with nonatypical endometrial hyperplasia. J Obstet Gynaecol Res.

[21] Schmidt T, Breidenbach M, Nawroth F, Mallmann P, Beyer IM, Fleisch MC, Rein DT (2009). Hysteroscopy for asymptomatic postmenopausal women with sonographically thickened endometrium. Maturitas.

[22] Famuyide AO, Breitkopf DM, Hopkins MR, Laughlin-Tommaso SK (2014). Asymptomatic thickened endometrium in postmenopausal women: malignancy risk. J Minim Invasive Gynecol.

